# Analysis of the Spatial and Temporal Evolution of Land Cover and Heat Island Effects in Six Districts of Chongqing’s Main City

**DOI:** 10.3390/s19235239

**Published:** 2019-11-28

**Authors:** Qin Lang, Wenping Yu, Mingguo Ma, Jianguang Wen

**Affiliations:** 1 Chongqing Engineering Research Center for Remote Sensing Big Data Application, School of Geographical Sciences, Southwest University, Chongqing 400715, China; langqin_joy@163.com (Q.L.); mmg@swu.edu.cn (M.M.); 2 Institute of Remote Sensing and Digital Earth, Chinese Academy of Sciences, Beijing 100101, China; wenjg@radi.ac.cn

**Keywords:** heat island effect, land cover, six districts of Chongqing’s main city, remote sensing

## Abstract

The urban heat island effect has always been one of the hottest issues in urban development. In this study, Landsat images from the summers of 2001, 2004, 2009, 2014 and 2018 were used to identify land cover type in six districts of Chongqing’s main city. Land cover was categorized as water, vegetation or impervious surface with the object-oriented method. Land surface temperature (LST) data was calculated with the atmospheric radiation transfer equation method, and was then divided into different heat island intensity grades. Next, the spatial and temporal changes in land cover type and heat island effect were analyzed in the six districts. Center migration analysis and heat island coefficients were used to quantitatively reflect the spatiotemporal evolution relationship between land cover and heat island effect. All six districts exhibited a trend of expanding impervious surface, with a 419.38% increase from 2001 to 2018, and shrinking vegetation, with a 17.81% decrease from 2001 to 2018. Also from 2001 to 2018, Yuzhong District had the most significant heat island effect, with a heat island coefficient 0.35 higher than the mean value of the whole study area. The impervious surface center migrated in different directions in each district. Both the direction and the corresponding velocity of the impervious surface and heat island centers were tightly correlated, with a correlation coefficient of 0.53. Relative heat island coefficients (the difference from the mean) of water ranged from −2.08 to −1.17 in different districts. That of impervious surface ranged from 1.60 to 1.93, and that of vegetation ranged from −0.22 to 1.09. The internal heterogeneity of land cover and heat island effect in Chongqing’s main city was huge. This study quantitatively analyzed the evolution of the heat island effect in the study area to help provide each district with some targeted suggestions for future urban planning.

## 1. Introduction

Urban environmental issues are at the intersection of three major problems in the world today: large population, limited resources and changing environment [1]. One of the important areas of urban environmental research is the urban heat island effect, which refers to the special microclimate formed under the influence of climate and human activity [2,3,4]. The urban heat island effect is caused by the imbalance of the urban environment and has become a major public hazard, seriously affecting the normal lives and health of urban residents [5,6,7,8,9]. There are roughly two research frameworks in urban heat research. The first is to assess the impact of urban planning on the environment using urban models that focus on possible mechanisms and future scenarios [10,11]. The second is to monitor urban heat in a city using observation data that describes the past [12,13,14,15,16,17]. These purposes go hand in hand because a deep understanding of past environmental conditions is necessary to develop better urban models to forecast the future. At present, the data used in urban heat monitoring primarily includes data from meteorological observation stations and remote sensing images [18]. Compared with the data from observation stations, remote sensing images are real-time, efficient and contain abundant information. Thus remote sensing images have become a significant data source for the study of urban heat islands [19,20,21,22,23,24]. Landsat images have a good spatial resolution and can provide abundant temperature information, and they are available over long time periods. Consequently, these images are suitable for monitoring the evolution of small-scale and fragmented landscape areas. Landsat images are often used to perform surface temperature inversion with mono-window algorithms and atmospheric radiation transfer Equations [25]. Due to the environmental changes between instances of image acquisition and how the inversion of temperature uses average atmospheric profile parameters, some errors arise. These errors make it difficult to directly use the inversion surface temperature for comparative analysis. To solve this problem, some researchers classify normalized surface temperature data into heat island grades before further analysis [26,27,28]. 

Land use/cover change (LUCC) is a field of research studying spatiotemporal changes in type and proportional area of impervious surface, vegetation, etc. These changes affect surface albedo, surface emissivity and surface roughness, as well as regional thermal environment [29]. A multitude of studies have focused on the forcing mechanism and LUCC prediction via models [30,31]. Nevertheless, it is also vital to monitor past LUCC in order to have a better understanding of the current regional thermal environment. Remote sensing is a powerful tool for extracting land cover information [32]. In remote sensing there are roughly two basic units of classification, the object and the pixel. Then there are various methods of classifying these units, including the maximum likelihood method, the support vector machine method, the decision tree method, the machine learning method [33,34], etc. Once the data has been extracted from the remote sensing images, there are two major methods to evaluate LUCC. The first is the statistical differences method, in which the absolute and relevant LUCC is extracted in different time thresholds. The second is the cross tabulation method, where all possible shifts in land use/cover are extracted [35]. With their efficiency, simple indicators like dynamic degree of single land use/cover type, amount of change and center of land use/cover are enough to evaluate LUCC without complicate shifts [36].

Chongqing’s main city is located in the eastern part of the Sichuan Basin, at the core of Chongqing municipality, which is known as the “furnace city”. The urban heat island effect is often studied here. In recent years Chongqing’s economy has developed rapidly, and so its land cover has also changed dramatically. The urbanization of the main city is the epitome of Chongqing’s recent development. Many studies have already shown that the urban heat island effect is very strong in the main city of Chongqing [37,38,39,40,41], but there has been a lack of analysis on its spatiotemporal evolution within each district of Chongqing’s main city. Some studies have shown that types of land cover and their transitions are important factors affecting the urban heat island effect [42,43]. The proportion of various land cover types and the speed of urban development varies across the different districts of Chongqing’s main city, but past research has blurred these distinctions by only considering Chongqing’s main city as a whole. In a larger-scale comparative study, other researchers have found that the type of land cover with the best mitigation for the urban heat island varies in different cities [44,45]. Because of the complex terrain and fragmented landscape in Chongqing’s main city, its thermal environment has great spatial heterogeneity. This makes it doubtful that the conclusions of a large-scale study can accurately represent the actual situation within each district. Hence, it is important to study whether or not the same land cover type differently impacts the urban thermal environment in each district, as well as how different these effects might be. By calculating the correlation between impervious surface area or impervious surface index and temperature, some studies have found that impervious surfaces greatly impact the thermal environment [46]. For instance, Wang et al. [47] selected 1000 random points in their study area to calculate the correlation between land surface temperature (LST) and impervious surface index. This method reflected the quantitative relationship between LST and impervious surface index, but it failed to describe the spatial relationship quantitatively. By accounting for both the rate and direction of spatiotemporal change, a quantitative analysis of the evolution of each district’s urban heat island in Chongqing’s main city can provide a more targeted scientific basis for future urban planning and development in each district, as well as a reference for urban heat island studies in other regions. 

In this paper, we took six districts in the main city of Chongqing as the basic research units: Yuzhong District, Jiangbei District, Nanan District, Shapingba District, Beibei District and Yubei District. We used Landsat series remote sensing images to classify land cover and to retrieve surface temperature, and then we analyzed the temporal and spatial evolution of land cover types and the heat island effect in the six districts. Next the shift in impervious surface and heat island centers, as well as their correlation, was analyzed in each district. Finally, we calculated the relative contribution of land cover types to the heat island through the heat island coefficients. The rest of this paper is structured as follows: material and methods; results and discussion; conclusions.

## 2. Material and Methods 

### 2.1. Study Area and Data

Chongqing, with a total area of 8.24 × 10^4^ km^2^, is located in the southwestern part of China. Chongqing’s administrative areas spans from Hubei and Hunan to Sichuan in the east-west direction, and from Shaanxi to Guizhou in the north-south direction. The main city of Chongqing is the political, economic, cultural, transportation and financial center of Chongqing municipality. It has a subtropical monsoon climate with an average annual precipitation of 1000~1800 mm and an average annual air temperature of 14.9~18.5 °C. In addition, Chongqing is a region that frequently experiences high temperatures and drought disasters [48].

**Figure 1:**
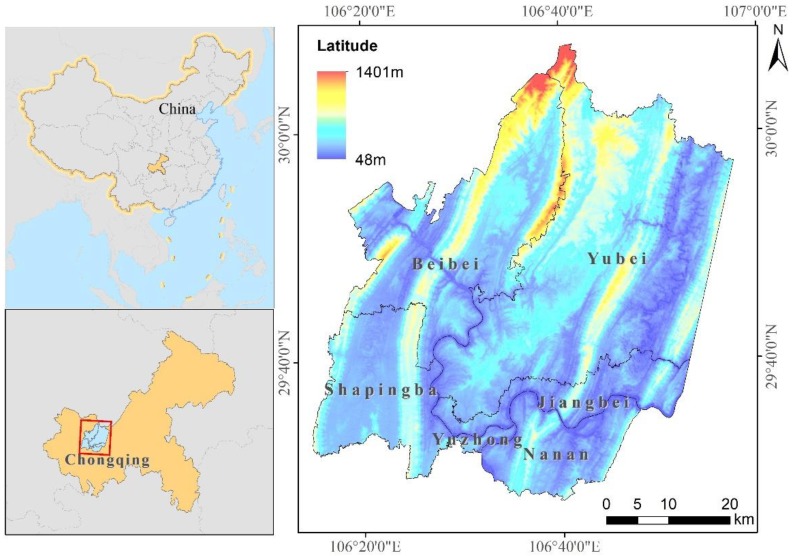
The study region and its location in China.

The study region lies from 106°14′17″ to 106°57′33″ E in longitude and from 29°27′39″ to 30°7′43″ N in latitude, with an area of 3028.24 km^2^ and an average elevation of 379.48 m. The study area lies at the intersection of Chongqing’s main city and the Landsat images with a rank number of 128039. This includes all of Yuzhong District, Shapingba District, Nan’an District, Beibei District and more than 90% of the Yubei and Jiangbei Districts (Figure 1). 

The data sources used in this study include Landsat images and verification points. Because the urban heat island effect is more pronounced in the summer [26], five Landsat series images from the summer were used (image details in Table 1). Among these five images, only two have more than 3% cloud cover, and the clouds in these two images are mainly distributed outside the study area. The optical and thermal images of Landsat 8 Operational Land Imager (OLI) and Landsat 5 Thematic Mapper (TM) were used in this study and were subjected to system radiation correction, ground control point geometry correction and terrain correction. They were provided by the Geospatial Data Cloud site of the Computer Network Information Center, Chinese Academy of Sciences. (http://www.gscloud.cn). The vector sample points used for the evaluation of the land cover classification data were obtained with reference to high-resolution images from Google Earth. The atmospheric profile parameters used to calculate brightness and temperature were obtained using the Atmospheric Correction Parameter Calculator (http://atmcorr.gsfc.nasa.gov/), which provides an automated method to derive atmospheric correction parameters needed for generating surface temperatures over large time periods and areas up to ±2–3 K [49].

### 2.2. Methodology

**Figure 2:**
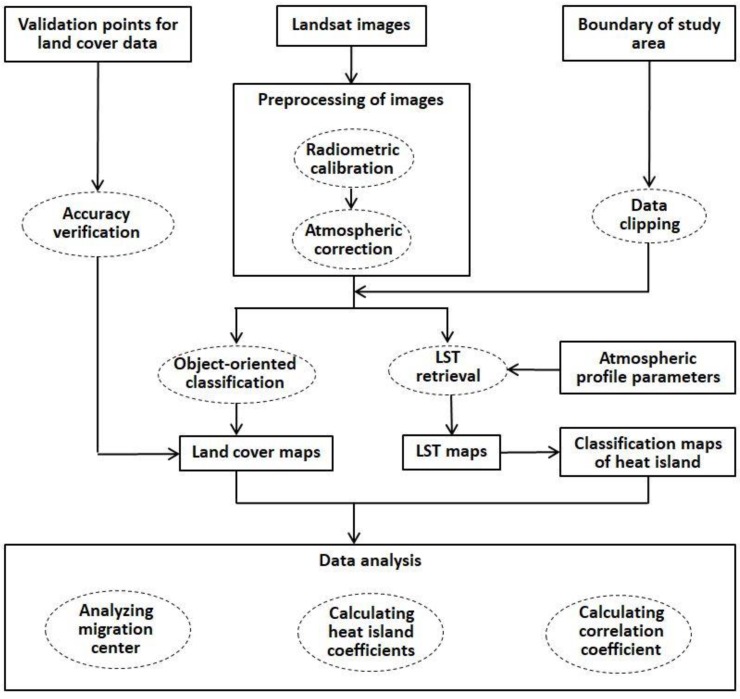
Representation of the methodological framework used in this study.

As shown in Figure 2, the Landsat images were preprocessed for land cover classification and LST retrieval. After that, LST data was divided into different heat island grades. Then the changes in land cover type, heat island effect, and the relationship between them were all analyzed for each district by calculating heat island coefficients and the migration of impervious surface and heat island centers.

#### 2.2.1. Image Preprocessing and Land Cover Analysis

**Figure 3:**
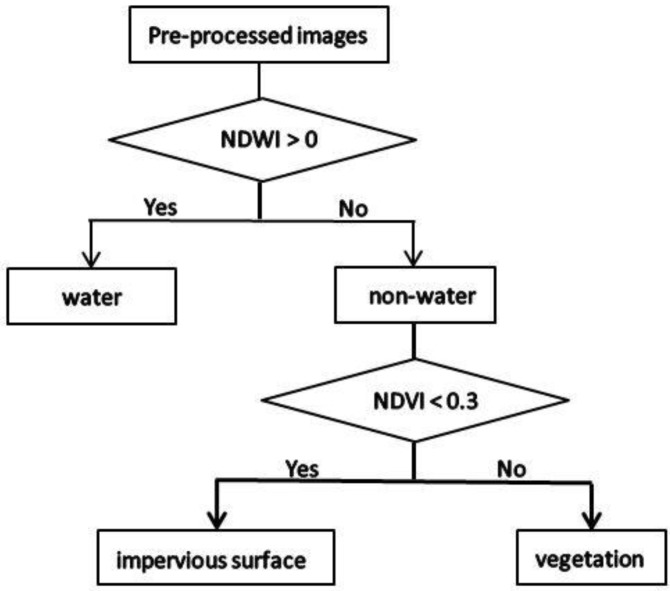
Classification rules of decision tree.

**Figure 4:**
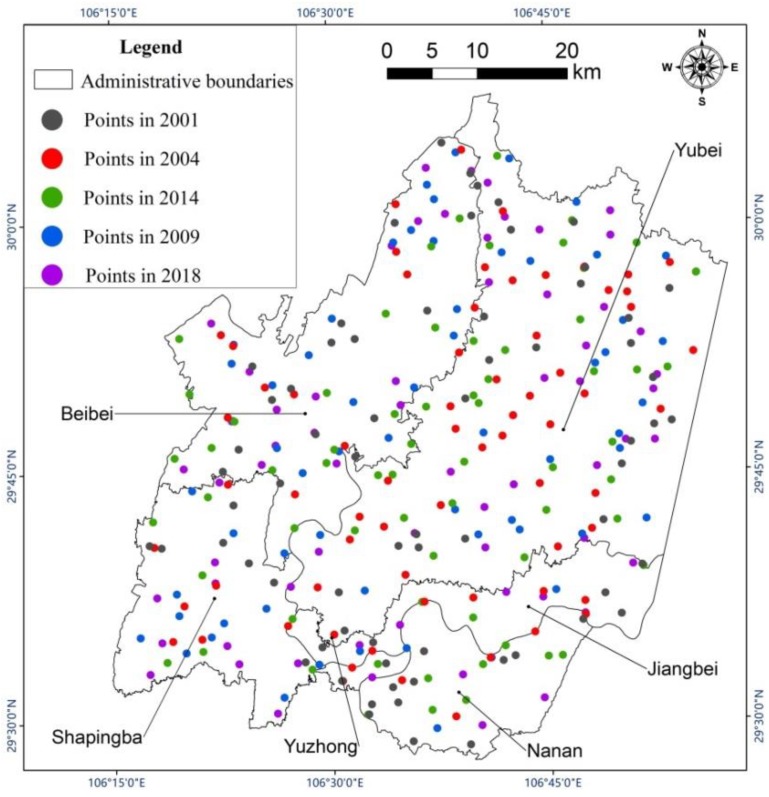
Distribution of verification points in different years.

We performed radiometric calibration and atmospheric correction on the satellite images used in this study. Because impervious surface (urban buildings, traffic roads, etc.) [47], vegetation (farmland, grassland, garden land, etc.) and water (rivers, reservoirs, etc.) have great and distinct effects on the thermal environment in urban areas, the classification system was constructed with these three types only. First, we merged pixels of pre-processed images into objects via multiresolution segmentation and spectral difference segmentation. Then, using the object-oriented decision tree classification method, the land cover types in the study area were classified according to the normalized difference water index (NDWI) and the normalized difference vegetation index (NDVI), which were calculated with Formulas (1) and (2). As shown in Figure 3, when the NDWI value was greater than 0, it was considered water, otherwise it was non-water. Non-water objects having an NDVI value greater than 0.3 were considered vegetation. If the object did not fit into either of the first two categories, it was considered impervious surface. Subsequently, as is shown in Figure 4, 328 total verification points were selected from Google Earth using a stratified random sampling method. This included 67 points for 2001 and 2004, 66 points for 2009 and 2018, and 62 points for 2014. For each one, we manually assessed the proper land cover type classification to aid in the evaluation of the accuracy of the automated land cover classification. The producer accuracy (PA), user accuracy (UA), overall accuracy (OA) and kappa coefficient were calculated with Equations (3) to (6). 

Our land cover classification system was not complicated. We assessed the classification differences in land cover across different time thresholds to obtain land cover change data. We used two indicators to represent land cover change: annual proportional change in a certain land cover and a certain land cover’s area proportional to the total land cover area. The two indicators were respectively calculated with Equations (7) and (8).

(1) $$NDWI=\;\frac{\rho \left(Green\right)-\rho \left(NIR\right)}{\rho \left(Green\right)+\rho \left(NIR\right)}$$

(2) $$NDVI=\;\frac{\rho \left(NIR\right)-\rho \left(Red\right)}{\rho \left(NIR\right)+\rho \left(Red\right)}$$

where ρ(Green) is the reflectance of the green band, ρ(NIR) is the reflectance of the near-infrared band, and ρ(Red) is the reflectance of the red band.

(3) $$PA_{i}=\;\frac{M_{i}}{P_{i}}$$

(4) $$UA_{i}=\;\frac{M_{i}}{U_{i}}$$

(5) $$OA=\;\frac{\sum_{i=1}^{3}M_{i}}{N}$$

(6) $$kappa=\;\frac{OA-\frac{\sum_{i=1}^{3}P_{i}*U_{i}}{N^{2}}}{1-\frac{\sum_{i=1}^{3}P_{i}*U_{i}}{N^{2}}}$$

(7) $$V_{i}=\frac{S_{bi}-S_{ai}}{Year_{b}-Year_{a}}$$

(8) $$K_{i}=\frac{S_{i}}{S_{total}}$$

where *PAi* is the producer accuracy of land cover *i* (1, 2 and 3 are used to represent different kinds of land cover), *M_i_* is the number of samples that were categorized into a type of land cover *i*, *P_i_* is the number of samples assigned to a particular land cover *i*, *UA_i_* is the user accuracy of land cover *i*, *U_i_* is the number of samples belonging to land cover *i* in the reference category, *OA* is the overall accuracy, *N* is the number of all samples, *kappa* is the kappa coefficient, *V_i_* is the annual change rate of land cover type *i* in the study area, $Year_{b}$ and $Year_{a}$ respectively represent the termination and start years of a certain time period (2001–2004, 2004–2009, 2009–2014 or 2014–2018), *S_bi_* and *S_ai_* respectively represent the area of land cover *i* in the end year and in the start year of a certain time period, *K_i_* is land cover *i*’s area proportional to the area of all land covers, *S_i_* represents the area of land cover *i* in a certain district or in the whole study area, *S_total_* represents the area of all land cover in a certain district or in the whole study area.

#### 2.2.2. LST Retrieval

The atmospheric radiation transfer equation, which enables surface temperature inversion for Landsat images, has good accuracy [26,50]. The influence of the atmosphere on surface thermal radiation was evaluated based on the atmospheric sounding data from satellite synchronous observation. The intensity of the surface thermal radiation can be obtained by subtracting the influence of the atmosphere from the total heat radiation measured by satellite sensors. In this study, we first calculated the blackbody radiance $B\left(T_{s}\right)$ using Equation ((9). To improve the accuracy of surface emissivity data, the method proposed by Qin et al. [50], which classifies the land cover types as water, vegetation and impervious surface, was used in this study. Then the emissivity of each land cover type was calculated respectively with Equations (10) through (12). Subsequently, we calculated vegetation coverage using Equations (13) through (15). Considering the local situation of the study area, we set 0.05 as the NDVI value for pixels of pure soil and 0.7 as the NDVI value for pixels of pure vegetation. Finally, the intensity of the surface thermal radiation was converted to the surface temperature with Equation (16), which is a simplified version of the Planck function [51,52,53].

(9) $$L_{\lambda }=\left[\varepsilon *B\left(T_{s}\right)+\left(1-\varepsilon \right)*L↓\right]*\tau +L↑$$

(10) $$\varepsilon_{water}=0.995$$

(11) $$\varepsilon_{veg}=0.9625+0.0614*Pv-0.04612*PV^{2}$$

(12) $$\varepsilon_{imp}=0.9589+0.086*Pv-0.0671*PV^{2}$$

(13) $$PV=\;\frac{NDVI_{i}-NDVI_{soil}}{NDVI_{veg}-NDVI_{soil}}\;\left(\mathrm{when}\;NDVI_{soil}\leq NDVI_{i}\leq NDVI_{veg}\right)$$

(14) $$PV=\;0\;(\mathrm{when}\;NDVI_{i}<NDVI_{soil})$$

(15) $$PV=\;1\;(\mathrm{when}\;NDVI_{i}>NDVI_{veg})$$

(16) $$T_{s}=\frac{K_{2}}{\left(ln\left(\frac{K_{1}}{B\left(T_{s}\right)}\right)+1\right)}$$

where $B\left(T_{s}\right)$ is blackbody radiance, $L_{\lambda }$ is thermal infrared radiation received by satellite sensors, L↑ is upward atmospheric radiation, L↓ is downward atmospheric radiation, ε is surface emissivity of a pixel, τ is atmospheric transmittance of the thermal infrared band, $\varepsilon_{water}$ is water’s surface emissivity, $\varepsilon_{veg}$ is vegetation’s surface emissivity,$\;\varepsilon_{imp}$ is impervious surface’s surface emissivity, *PV* is vegetation coverage, $NDVI_{i}$ is the *i*th pixel’s NDVI value on the image, $NDVI_{soil}$ is the NDVI value for pixels of pure soil, $NDVI_{veg}$ is the NDVI value for pixels of pure vegetation, $T_{s}$ is land surface temperature, $K_{1}$ and $K_{2}$ are calibration coefficients of Landsat images (unite: °C).

#### 2.2.3. Analysis of Heat Island Grades and Calculation of Heat Island Coefficients

Using Equation (17), we normalized the LST values to be between 0 and 1. Then we used the mean-standard deviation calculated with Equations (18) and (19) to classify the heat island grades [54], and we assigned different heat island grades ranging from 1 to 6 in order of increasing heat (Table 2). These weights were then involved in calculating the heat island center as well as the heat island coefficients. These metrics quantitatively reflected the spatiotemporal evolution of the heat island and its influence on the thermal environment of the various land cover types in each district. The method used to analyze change in heat island grades was replicated for land cover change analysis. We mainly used two indicators, the annual change rate of each heat island grade and the proportion of each heat island grade relative to all others. The indicators were respectively calculated with Formulas (7) and (8).

(17) $$T_{norm\_i}=\frac{T_{s\_i}-T_{s\_min}}{T_{s\_max}-T_{s\_min}}$$

(18) $$u=\frac{\sum_{i=1}^{n}T_{\mathrm{norm}\_i}}{n}$$

(19) $$std=\frac{\sum_{i=1}^{n}{\left(T_{\mathrm{norm}\_i}-u\right)}^{2}}{n-1}$$

where *T_norm_i_* is the normalized result for the *i-*th pixel in the LST raster map, *T_s_i_* is the LST of the *i*th pixel, *T_s_min_* is the minimum pixel value in the LST raster map, *T_s_max_* is the maximum pixel value in the LST raster map, u is the mean of all normalized pixel values, *std* is the standard deviation of all normalized pixel values.

The heat island coefficients were calculated according to the area and the corresponding weight of heat island grades (Table 2). The relative heat island coefficients were also calculated for each land cover type in each district by subtracting the local heat island coefficient from the mean heat island coefficient for all land cover types in all districts within the study area. These were used to quantitatively evaluate the relative contribution of the land cover in different districts to the thermal environment. A relative heat island coefficient greater than 0 suggests that the area contributes to the heat island effect, and a value less than 0 suggests that the area weakens the heat island effect. The greater the absolute value, the stronger the effect on heat island effect. The heat island coefficients for each land cover type in the different districts were calculated as follows:

(20) $$I_{ja}\;=\frac{\sum_{i=1}^{6}S_{ai}*W_{i}}{\sum_{i=1}^{6}S_{ai}}$$

where $I_{ja}$ is the heat island coefficient of land cover *a* in district *j*, $S_{ai}$ is the area of land cover *a* considered to have heat island grade *i* in district *j*, $W_{i}$ is the weight value of heat island *i*.

#### 2.2.4. Center Migration and Correlation Analysis

In this study, we calculated the center of thermal grade patches and impervious surfaces. The center of impervious surface was the geometric center of all patches in each district that were classified as impervious surface. The center of the heat island was calculated as follows:

(21) $$X_{j}=\frac{\sum_{i=1}^{6}X_{i}{*W}_{i}}{\sum_{i=1}^{6}W_{i}}$$

(22) $$Y_{j}=\frac{\sum_{i=1}^{6}Y_{i}{*W}_{i}}{\sum_{i=1}^{6}W_{i}}$$

where $X_{j}$ is the center of the heat island’s abscissa coordinate in district *j*, $Y_{j}$ is the center of the heat island’s ordinate coordinate in district *j*, $X_{i}$ is the abscissa coordinate center of heat island *i*, $Y_{i}$ is the ordinate coordinate center of heat island *i*, and $W_{i}$ is the weight value of heat island *i*.

In order to further analyze the correlation between the direction and degree of impervious surface and heat island center migration, the east-west migration rate, *V_X_*, the north-south migration rate, *V_Y_* and the total migration rate, *V* (m/year), were determined separately. The Pearson correlation coefficients (denoted as $r_{PQ}$) of *V_X_*, *V_Y_* and V of the shifting impervious surface and heat island center were each calculated to judge the degree of correlation between them. With four time spans (2001 to 2004, 2004 to 2009, 2009 to 2014, 2014 to 2018), three kinds of rates (*V_X_*, *V_Y_* and *V*) and six districts, there were 72 (4 × 3 × 6) groups of center migration rates for which to calculate $r_{PQ}$. The $r_{PQ}$, *V_X_*, *V_Y_* and V were calculated as follows:

(23) $$V_{X}=\frac{X_{b}-X_{a}}{Year_{b}-Year_{a}}$$

(24) $$V_{Y}=\frac{Y_{b}-Y_{a}}{Year_{b}-Year_{a}}$$

(25) $$V=\sqrt{V_{X}^{2}+V_{Y}^{2}}$$

(26) $$r_{PQ}=\frac{\sum_{i=1}^{72}(P_{i}-\bar{P})*(Q_{i}-\bar{Q})}{\sqrt{\sum_{i=1}^{72}{\left(P_{i}-\bar{P}\right)}^{2}}*\sqrt{\sum_{i=1}^{72}{\left(Q_{i}-\bar{Q}\right)}^{2}}}$$

where $Year_{b}$ and $Year_{a}$ respectively represent the termination and start years of a certain time period (2001–2004, 2004–2009, 2009–2014 or 2014–2018), *X_b_* and *Y_b_* respectively represent the coordinates of the center in the east-west and north-south directions in the end year of a certain time period (unite: m), *X_a_* and *Y_a_* respectively represent the coordinates of the center in the east-west and north-south directions in the start year of a certain time period (unite: m), *P_i_* and *Q_i_* respectively represent the *i-*th group of impervious surface and heat island center migration rates, $\bar{P}$ and $\bar{Q}$ respectively represent the average impervious surface and heat island center migration rates.

## 3. Results and Discussion

### 3.1. Land Cover Changes

**Figure 5:**
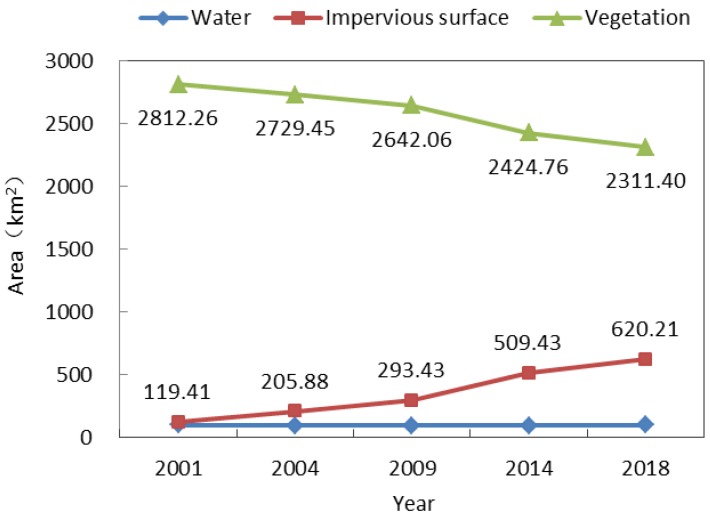
Changes in land cover type across the research area.

**Figure 6:**
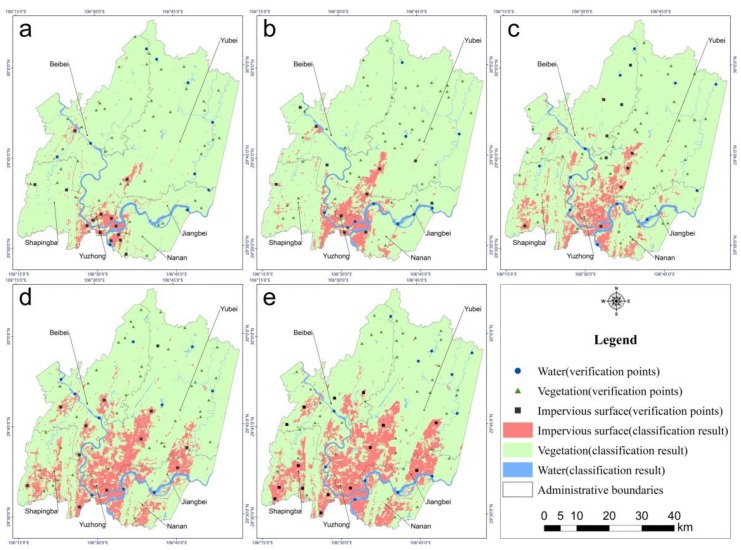
Distribution of land cover and verification points in 2001 (**a**), 2004 (**b**), 2009 (**c**), 2014 (**d**), and 2018 (**e**), respectively.

We used verification points to evaluate the accuracy of land cover type classification. As demonstrated in Table 3, kappa coefficients were greater than 0.80 and overall accuracy was greater than 0.90 in 2001, 2004, 2009, 2014 and 2018. As shown in Figure 5, the proportional area of water cover was always the smallest, and its area did not change much from 2001 to 2018. In contrast, the impervious surface area gradually increased from 119.41 km^2^ in 2001 to 620.21 km^2^ in 2018, with the highest average annual growth rate occurring from 2009 to 2014 at 43.20 km^2^/year. Finally, the proportional area of vegetation was always the largest, but it decreased from year to year, mostly in accordance with the increase in impervious surface area. Figure 6 shows the spatial distribution of land cover types in 2001, 2004, 2009, 2014 and 2018. 

**Figure 7:**
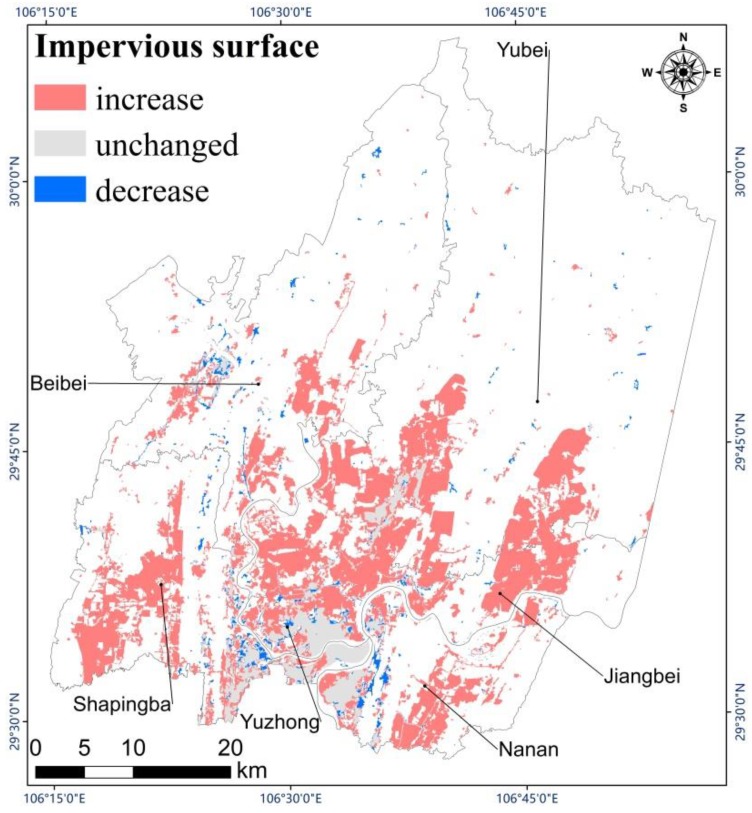
Change of impervious surface from 2001 to 2018.

Impervious surface had a tendency to expand in both directions along a northeast axis. Although the impervious surface expanded rapidly toward the northeast, the northern part of Chongqing was still mainly covered by vegetation. Figure 7 shows the total spatial variation of impervious surface during the 17 years from 2001 to 2018. The unchanged areas are mainly distributed in and around Yuzhong District. Few areas decreased in impervious surface, and these were mainly distributed around the Yuzhong and the Beibei Districts. The four other districts tended to increase in impervious surface over the study period. By observing Google Earth Images, we found that most impervious surfaces in the study region contained buildings and that construction was the main reason why vegetation decreased and impervious surface increased.

**Figure 8:**
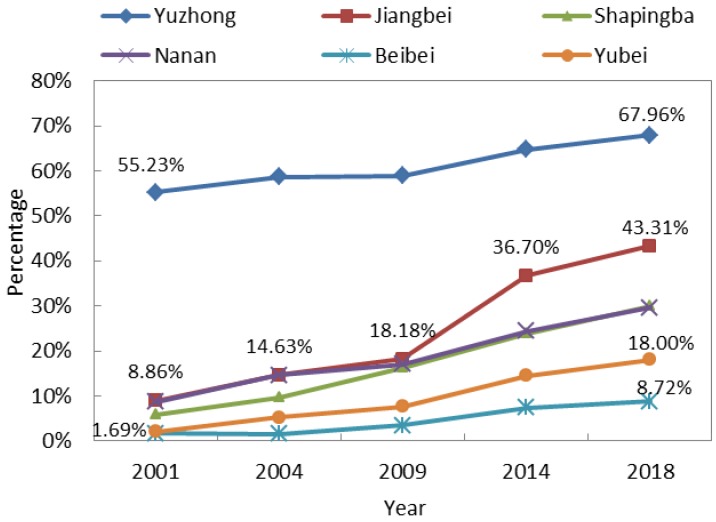
Variation in proportional area of impervious surface in different districts.

Because the expansion of impervious surface was tremendous and mainly caused by human activities in the study area, the change in proportional area of impervious surface from 2001 to 2018 was further analyzed. We calculated the variation in proportional area of impervious surfaces across the different districts (Figure 8). In order of greatest to lowest proportion of impervious surface, the six districts can be ranked as follows: Yuzhong District, Jiangbei District, Nanan District, Shapingba District, Yubei District and Beibei District. The impervious surface area proportion increased in all districts from 2001 to 2018, but the proportional and its temporal evolution varied greatly among the six districts. In the Yuzhong District, the proportional impervious surface area had been over 55% since 2001. By 2018 it had gradually increased to 67.69%. The proportional area of impervious surface was especially high in the Yuzhong District because it has been the political and commercial center in Chongqing for a while, at least since 2001. By sharp contrast, the proportional area of impervious surface was always lowest in the Beibei District, though it increased from 1.69% in 2001 to 8.72% in 2018. This low proportion is attributed to its great distance from Chongqing’s main city center, as well as its reputation as Chongqing’s “backyard”. Generally, the most conspicuous increase in proportional area of impervious surface occurred from 2009 to 2014 in all six districts, and the increase was especially remarkable in the Jiangbei District, where the proportion increased 18.52% more than the other districts. Governmental decisions vastly influenced this phenomenon. On June 18, 2010, the “Liangjiang New Area” was officially established as an important part of the government plan that contributed to rapid urban development in the Jiangbei District and beyond. From the above analysis, it is apparent that Chongqing’s main city experienced outstandingly rapid urban development from 2001 to 2018 and that the development differed notably among the districts. Thus it is crucial to study urban thermal environment based on each district in Chongqing’s main city.

### 3.2. Changes in the Heat Island Effect

**Figure 9:**
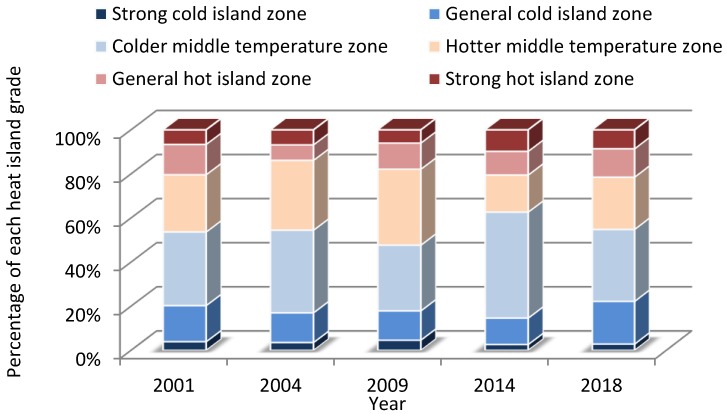
Proportional changes in heat island grade throughout the study area.

Figure 9 shows the proportional area of heat island effect in the whole study area. The proportional area of the strong cold island zone trended downward, from 3.92% in 2001 to 2.86% in 2018. The proportional area of the general cold island zone also gradually decreased, from 16.38% in 2001 to 11.98% in 2014, though it rose to 19.35% in 2018. As for the proportional area of the strong heat island zone, it first increased from 6.63% in 2001 to 9.77% in 2014, and then it decreased slightly to 8.59% in 2018. The other proportional areas, of the colder middle temperature zone, the hotter middle temperature zone and the general heat island zone, did not show a consistent trend. In 2009, the dominant heat island grade was the hottest middle temperature zone. In conclusion, the entire study area had an enhanced heat island effect, with the area of the strong heat island zone increasing and the area of the strong cold heat island zone decreasing from 2001 to 2018, both exerting great influence on the local environment.

**Figure 10:**
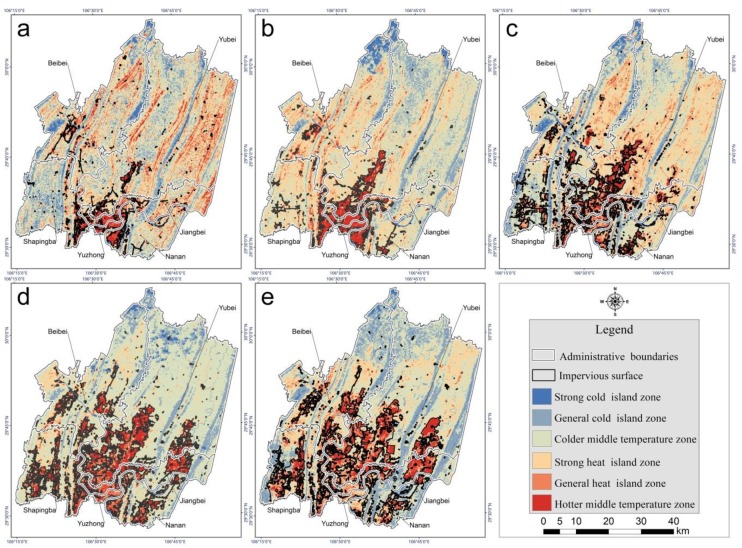
Classification of heat island strength in 2001 (**a**), 2004 (**b**), 2009 (**c**), 2014 (**d**), and 2018 (**e**).

As shown in Figure 10, the spatial distribution of both the strong and the general heat island zones had a tendency to expand in both directions along the northeast axis, which also occurred for impervious surfaces. The southern part of the study area was covered mainly by impervious surface and showed an increasingly strong urban heat island in accordance with the impervious surface expansion from 2001 to 2018. However, the northern part of the study area was mainly covered by vegetation and lacked a trend of gradually increasing heat during the study period, yet it had a hotter thermal environment in 2001 and in 2009 than in the other three years. This is because Chongqing experienced serious drought disasters in 2001 and 2009 [55,56] which impacted healthy vegetation growth and heightened the thermal environment.

Through the analysis of the heat island effect in the different districts, the following conclusions were drawn: Yuzhong District’s heat island effect was the most significant among all the districts, and its dominant heat island grade was strong heat island zone in 2001 and 2004, though its heat island effect gradually increased in the later period. In 2009 and 2014, Yuzhong District’s dominant heat island grade transitioned into general heat island zone, and then in 2018 it gained hotter middle temperature zone status. The dominant heat island grades of the other districts were hotter or colder middle temperature zones from 2001 to 2018. Statistics regarding the average proportional area of extreme heat island grades, including the strong cold and strong heat island zones, showed that in 2001, 2004, 2009, 2014 and 2018, Yuzhong District had the largest proportional area of strong heat island zone (29.92%), followed by Jiangbei District (16.60%). Beibei District had the smallest proportional area of strong heat island zone (4.00%). Yuzhong District also had the largest proportional area of strong cold island zone (14.13%), followed by Nanan District (7.06%). Yubei District had the smallest proportional area of strong cold island zone (1.94%). 

Overall, the distribution of heat island grades was extremely unbalanced in the Yuzhong District, with too high a proportion of strong heat and strong cold island zones. This greatly reduced the comfort of residents and was not conducive to good health. Although all these districts belong to Chongqing’s main city, the thermal environment of the Beibei District was better than that of the Yuzhong District. It will be vital for the local government to adjust urban planning practices in Chongqing’s main city in order to develop the city in a more balanced and healthier way in the future.

### 3.3. Impervious Surface and Heat Island Center Migration

**Figure 11:**
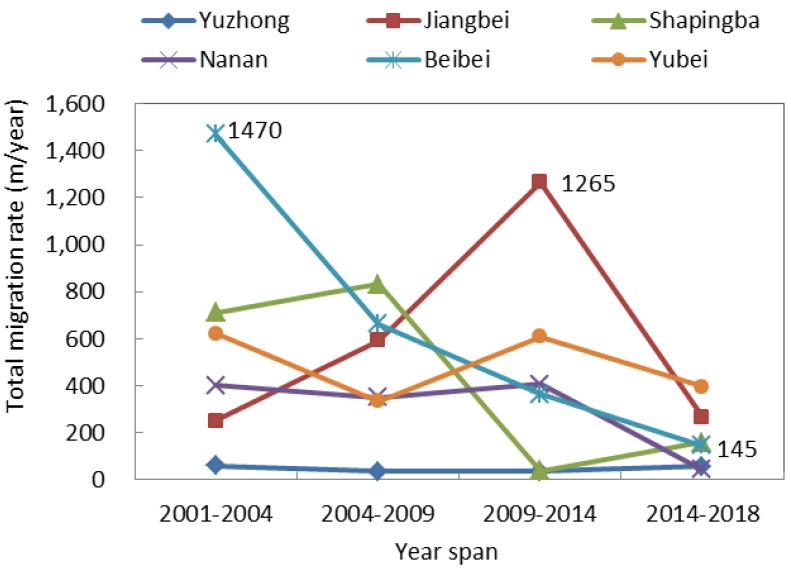
Total impervious surface center migration rate.

**Figure 12:**
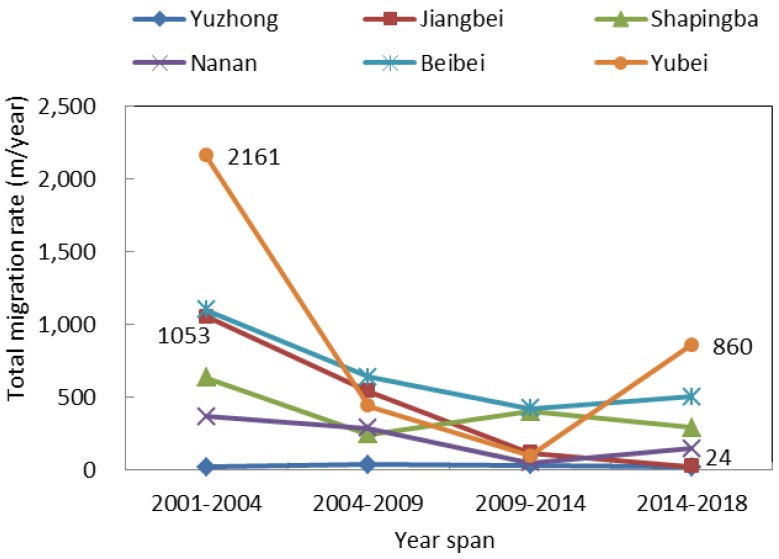
Overall heat island center migration rate.

We further analyzed the impervious surface center migration using the five-stage impervious surface data, as well as the heat island strength classification data. In terms of the impervious surface center migration rate, the total migration rate in Beibei District showed a continuous downward trend, from 1470 m/year in 2001–2004 to 145 m/year in 2014–2018. The total migration rate in Jiangbei District increased sharply from 2001 to 2014 and dropped sharply to 267 m/year from 2014 to 2018. The overall migration rate in Yuzhong District was very slow, never higher than 60 m/year. In the remaining districts, the overall migration rate varied irregularly (Figure 11). The difference in migration rates among these six districts was mainly attributed to urban planning. For example, construction has saturated the Yuzhong District, and local government has been focusing on expanding the Jiangbei District since 2010, so the migration rate was very slow in the Yuzhong District but extremely fast in the Jiangbei District. As shown in Figure 12, the overall migration rate showed a continuous downward trend in Jiangbei District, from 1053 m/year in 2001–2004 and 24 m/year in 2014–2018. The overall heat island center migration rate fluctuated the most in the Yubei District, where the highest rate occurred from 2001–2004 (2161 m/year) and the lowest from 2009–2014 (97 m/year). The lowest overall heat island center migration rate appeared in Yuzhong District, never higher than 38 m/year.

**Figure 13:**
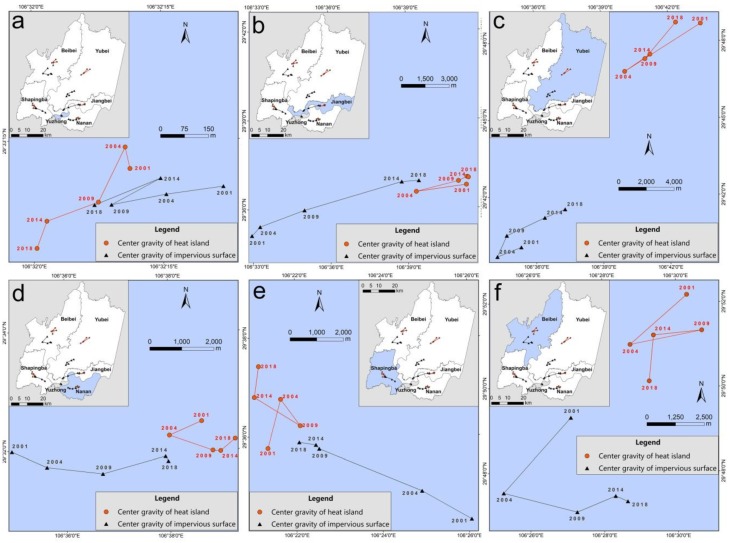
Impervious surface and heat island center migration in Yuzhong (**a**), Jiangbei (**b**), Yubei (**c**), Nanan (**d**), Shapingba (**e**), and Beibei Districts (**f**).

The locations of the various heat island centers were not highly consistent with that of the impervious surface centers (Figure 13). Especially for the Beibei and Yubei Districts, their heat island centers were located to the northeast of the impervious surface center, further than in the other four districts. The proportional impervious surface area was always lowest in the Beibei and Yubei Districts, never exceeding 18%. Hence, the influence of the impervious surface on the heat island center was weaker in these districts. The local center migration directions were consistent between impervious surface and heat island in most years, but each district had a different direction. In the Yuzhong District, migration occurred mainly toward the southwest. Jiangbei District’s centers continuously moved toward the northeast, Shapingba District’s centers continuously moved toward the northwest and Nanan District’s centers continuously moved westward. Beibei and Yubei Districts’ centers moved in multiple direction, toward the southwest from 2001 to 2004 and eastward from 2004 to 2018. 

In short, the overall impervious surface center migration direction was outward radiation expansion. In order to quantitatively analyze the correlation between direction and degree of center migration for both the impervious surface and the heat island, a Pearson correlation coefficient was calculated (0.53) using the east-west migration rate, the north-south migration rate and the total migration rate as data sources. Thus it became clear that the direction and degree of impervious surface center migration greatly influenced that of the heat island.

### 3.4. Contribution of Different Land Cover Types to Heat Island Effect 

The average weight of all heat island grades in theory was 3.5. However, the actual mean heat island coefficient of the entire study region from 2001 to 2018 was 3.54, slightly higher than the theoretical average. Although almost half of the northern study areas were mostly covered by vegetation, the rapidly increasing impervious surface area had a stronger influence on thermal environment and even augmented the heat island effect throughout the entire study region. In this study, we used 3.54 as a reference to quantitatively evaluate how the thermal environment of each district compared to the rest, depending on the effects of proportional land cover type. Table 4 shows that water had the most significant cooling effect (values less than 0), and impervious surface had the most significant warming effect (values greater than 0). The vegetation in all five districts besides Yuzhong had a cooling effect on the thermal environment.

There was a positive correlation between the cooling effect of vegetation and the proportional area of vegetation. The proportional area of vegetation was smallest in the Yuzhong District at 13.70%. The vegetation in this district had the weakest mitigation effect on the heat island coefficient, even enhancing the heat island coefficient, which was 1.09 greater than the study-wide average. Beibei District had the largest proportional area of vegetation (92.97%) and the greatest mitigation effect on the heat island coefficient, which was 0.22 lower than the study-wide average. Neither water nor impervious surface proportional area greatly influenced heat island coefficients on their own. Instead, the influence on heat island coefficients was determined by proportional area of both impervious surface and water. For example, heat island coefficient mitigation was most significant in the Nanan District at 2.08 lower than the study-wide average, but the proportional area of water was only 8.65%. In contrast, the proportional area of water was highest in the Yuzhong District at 25.19%, but its average heat island coefficient was 1.65 higher than the study-wide average. Although the proportional area of water was highest in Yuzhong District, the proportional area of impervious surface was also at its highest (61.11%), while the proportional area of impervious surface was only 18.87% in Nanan District. The impervious surface of the study region was mainly constructed along the river, and its density greatly affected the mitigation effect of water on heat island coefficients. Hence, in Chongqing’s main city, planning a rational design that designates where certain land cover types should go, especially for water and impervious surface, can reduce heat island effects when it is infeasible to adjust the proportions of land cover area instead.

## 4. Conclusions

Based on Landsat images of six districts in the main city of Chongqing that were taken from 2001 to 2018, this study explored the spatial and temporal evolution of land cover and heat island effect, as well as the relationship between them throughout the study time frame. This study showed that the six districts experienced notable land cover changes from 2001 to 2018, with the general trend involving the expansion of impervious surface and the corresponding reduction of vegetation. Also, the degree of change in land cover was found to be distinct in the various districts. According to a spatiotemporal analysis of heat island grades, we found that the spatiotemporal distribution and variation of impervious surface was highly coincident with that of the heat island grades which implied a strong heat island effect. Therefore, we could infer that impervious surface was one of the most important factors contributing to the urban heat island effect in Chongqing’s main city. Further analysis of the heat island effect in the six districts showed that the main heat island intensity levels in Yuzhong District throughout the study time frame were the strong and general heat island zones. In contrast, the other five districts were mainly the hotter middle and colder middle temperature zones. In order to better understand how impervious surface influenced the heat island effect, we quantitatively analyzed the correlation of center migration direction and rate between heat island and impervious surface. A correlation coefficient of 0.53 showed that the direction and degree of the impervious surface center greatly influenced that of the heat island center. Though impervious surface was a significant element that affected the heat island effect in Chongqing’s main city, it was also important to consider the influence of other land cover types. Hence, we analyzed the effects of all land cover types based on heat island coefficients, and we found that vegetation proportional area negatively influenced the heat island coefficients. Further, the proportional area of impervious surface and water also jointly influenced the heat island coefficients, even though their own influence was not significant.

In light of our findings in the broader context of similar studies, we offer several targeted suggestions for future urban planning efforts in the various districts of Chongqing’s main city in order to mitigate the urban heat island effect and to encourage more sustainable development. With the highest impervious surface density and the most significant urban heat island effect, Yuzhong District especially needs to ameliorate its thermal environment. Because of Yuzhong District’s crucial political and economic role in the Chongqing municipality, it is not practical to transform the impervious surface area into vegetation. However, urban planners can optimize limited urban green space by making intentional decisions regarding distances from trees to buildings, tree species selection, and shaping the height, form and structure of the urban tree canopy [57,58,59], etc. At the same time, urban planners can gradually transfer part of Yuzhong District’s urban function to proximate districts. With its undeveloped areas, Beibei District still contains much flexibility for urban design plans. It will be feasible to create a favorable urban micro-climate here by installing a rational urban configuration that accounts for street orientation, sky view factor, building density and height [60,61,62,63,64,65], etc. Currently, Jiangbei District is experiencing rapid urban development, so it will be especially critical to use environmentally-friendly building materials throughout this development. For example, conventional concrete can be replaced with permeable concrete or porous masonry to reduce the warming effects of impervious surface [10,11]. The other three districts are in intermediate developmental stages, so they ought to take their cues from both Beibei and Jiangbei Districts. In addition to our practical targeted suggestions for the districts, our study is also valuable for related research efforts. For instance, our quantitative methods of analyzing heat island coefficients and center migration can be applied to other regions where the urban heat island is prominent. Secondly, our district-level study provides a framework for how to design targeted micro-level studies for different districts in other urban models. Perhaps further studies in Yuzhong District can focus on determining the ideal thresholds for parameters like distance from tree to building, tree species, and height, form and structure of the tree canopy, etc. We might suggest that future studies in Beibei District focus on creating an environmentally-minded urban configuration.

There is still more to be studied. On one hand, we may compare micro-level studies in other regions to this one in an effort to provide more direction for improving the thermal environment in Chongqing’s main city. However we should keep in mind that conclusions from studies in other regions can serve as a reference, but they may not be greatly consistent with the actual situation in Chongqing’s main city. Consequently, we should continue to study Chongqing’s main city at a micro-level to answer these localized questions. On the other hand, here we only studied the influence of land cover types on urban heat island effects, so the influence of other factors, such as global warming, regional climate changes, population distribution, and economic development, etc., on the urban heat island may be worth further study.

## Figures and Tables

**Table 1:** Details of Landsat images used in this study.

Satellite	Row	Path	Acquisition Date	Spatial Resolution (Multispectral/Thermal Infrared)	Cloud Cover (%)
Landsat 5	128	39	2001–07–17	30 m/120 m	0.01
Landsat 5	128	39	2004–07–25	30 m/120 m	0.49
Landsat 5	128	39	2009–08–24	30 m/120 m	11.41
Landsat 8	128	39	2014–08–06	30 m/100 m	2.92
Landsat 8	128	39	2018–09–02	30 m/100 m	17.79

**Table 2:** Classification criteria for heat island grades.

Name	Classification Standard	Weight
strong cold island zone	[0,u − 1.5 ∗ *std*)	1
general cold island zone	[*u* − 1.5 ∗ *std*,*u* − 0.75 ∗ *std*)	2
colder middle temperature zone	[*u* − 0.75 ∗ *std*,*u*)	3
hotter middle temperature zone	[*u*,*u* + 0.75 ∗ *std*)	4
general heat island zone	[*u* + 0.75 ∗ *std*,*u* + 1.5 ∗ *std*)	5
strong heat island zone	[*u* + 1.5 ∗ *std*,1]	6

**Table 3:** Evaluation of land cover classification.

Evaluation Indicators	2001	2004	2009	2014	2018
**Kappa coefficient**	0.89	0.84	0.81	0.91	0.86
**Overall accuracy**	0.94	0.93	0.91	0.95	0.92

**Table 4:** Relative heat island coefficients of every land cover type in the six districts.

District	Water	Vegetation	Impervious Surface	Mean of All Land Cover Types
**Yuzhong**	−1.65	1.09	1.67	0.37
**Jiangbei**	−2.05	−0.01	1.92	−0.05
**Shapingba**	−1.17	−0.14	1.64	0.11
**Nanan**	−2.08	−0.22	1.60	−0.23
**Beibei**	−1.29	−0.22	1.65	0.05
**Yubei**	−1.88	−0.08	1.93	−0.01
**Mean of each land cover type**	−1.95	0.04	1.91	
